# Unveiling Inter- and Intra-Patient Sequence Variability with a Multi-Sample Coronavirus Target Enrichment Approach

**DOI:** 10.3390/v16050786

**Published:** 2024-05-15

**Authors:** Sara Lado, Jakob Thannesberger, Kathrin Spettel, Jurica Arapović, Bibiana I. Ferreira, Marialuisa Lavitrano, Christoph Steininger

**Affiliations:** 1 Division of Infectious Diseases and Tropical Medicine, Department of Medicine 1, Medical University of Vienna, 1090 Vienna, Austria; sara.lado@meduniwien.ac.at (S.L.); jakob.thannesberger@meduniwien.ac.at (J.T.); 2 Division of Clinical Microbiology, Department of Laboratory Medicine, Medical University of Vienna, 1090 Vienna, Austria; kathrin.spettel@meduniwien.ac.at; 3 Division of Biomedical Science, University of Applied Sciences, FH Campus Wien, 1100 Vienna, Austria; 4 Department of Medical Biology, School of Medicine, University of Mostar, Bijeli Brijeg b.b., 88000 Mostar, Bosnia and Herzegovina; 5 Faculty of Medicine and Biomedical Sciences, University of Algarve, Campus de Gambelas, Edf. 2, 8005-139 Faro, Portugal; bibiana.i.ferreira@gmail.com; 6 Algarve Biomedical Center Research Institute, Campus de Gambelas, Edf. 2, lab 3.67, 8005-139 Faro, Portugal; 7 School of Medicine and Surgery, Milano-Bicocca University, 20900 Monza, Italy; marialuisa.lavitrano@unimib.it; 8 Karl-Landsteiner Institute for Microbiome Research, Medical University of Vienna, 1090 Vienna, Austria

**Keywords:** variants, pandemic, metagenomics, virus, target enrichment, frequency, high throughput, COVID-19

## Abstract

Amid the global challenges posed by the COVID-19 pandemic, unraveling the genomic intricacies of SARS-CoV-2 became crucial. This study explores viral evolution using an innovative high-throughput next-generation sequencing (NGS) approach. By taking advantage of nasal swab and mouthwash samples from patients who tested positive for COVID-19 across different geographical regions during sequential infection waves, our study applied a targeted enrichment protocol and pooling strategy to increase detection sensitivity. The approach was extremely efficient, yielding a large number of reads and mutations distributed across 10 distinct viral gene regions. Notably, the genes Envelope, Nucleocapsid, and Open Reading Frame 8 had the highest number of unique mutations per 1000 nucleotides, with both spike and Nucleocapsid genes showing evidence for positive selection. Focusing on the spike protein gene, crucial in virus replication and immunogenicity, our findings show a dynamic SARS-CoV-2 evolution, emphasizing the virus–host interplay. Moreover, the pooling strategy facilitated subtle sequence variability detection. Our findings painted a dynamic portrait of SARS-CoV-2 evolution, emphasizing the intricate interplay between the virus and its host populations and accentuating the importance of continuous genomic surveillance to understand viral dynamics. As SARS-CoV-2 continues to evolve, this approach proves to be a powerful, versatile, fast, and cost-efficient screening tool for unraveling emerging variants, fostering understanding of the virus’s genetic landscape.

## 1. Introduction

Since the start of the COVID-19 pandemic, society has been engaged in a persistent race for the development of countermeasures against the ever-evolving SARS-CoV-2 virus. This dynamic mirrors the concept of the Red Queen hypothesis [1], in which the host and the parasite engage in a continuous evolutionary arms race, adapting to each other, ultimately shaping their fitness and survival traits in response to this coexistence. The emergence of novel viral variants with mutations that may confer higher transmissibility and resistance to immunity from vaccines or previous infections increases the stakes in this race [2].

The last pandemic prompted an unprecedented global response, marked by vigilant scientific surveillance aimed at tracking the evolution of SARS-CoV-2 in near real time [3]. From the millions of documented COVID-19 cases worldwide, over 16 million whole genome sequences have been generated and shared in the publicly accessible GISAID database (www.gisaid.org; accessed on 30 January 2024). This extensive genomic surveillance has been possible through advances in pathogen sequencing technology, enabling the identification of specific genetic markers associated with emerging virus variants and their potential to impact viral pathogenicity and immune evasion, particularly those affecting the spike (S) protein and the Receptor Binding Domain (RBD; [4]).

Because of selection pressure and mutation rates, allele frequencies within the SARS-CoV-2 population change over time, resulting in the preferential selection of genotypes that confer a fitness advantage. While early SARS-CoV-2 sequences showed limited genetic diversity, owing primarily to neutral evolution, it is now estimated that the virus is evolving at a moderate rate in comparison with other RNA viruses [5]. As the pandemic has progressed, numerous novel variants have emerged, some of which exhibit concerning patterns of local prevalence, prompting national healthcare authorities such as the U.S. Center for Disease Control (CDC) and the U.K. Health Security Agency to establish classification schemes for these variants, including Variants of Concern (VOC). These VOCs, including B.1.1.7 (Alpha), B.1.351 (Beta), P.1 (Gamma), B.1.617.2 (Delta;), or, more recently, B.1.1.529 (Omicron) among others, have generated greater public interest and highlighted the importance of early detection of emerging variants because of the associated increased risks.

While surveillance programs have aided the early detection of VOCs, some of which have since gained dominance in large geographic regions, and even globally, genetic virus surveillance has emerged as the most important tool for predicting future trends in the COVID-19 pandemic. In the clinical setting, various methods such as quantitative real-time PCR (qRT-PCR) and antigen–antibody-based serological assays are used to detect SARS-CoV-2, but these approaches typically do not assess genomic sequence variation.

In contrast, NGS technology strategies, which enabled early sequencing of the SARS-CoV-2 genome, are a viable option for screening viral sequence diversity. In particular, sophisticated NGS targeting approaches, such as capture-based enrichments, can enable early sequencing of the SARS-CoV-2 genome with high sensitivity [6]. By targeting specific genome regions relevant to the research objectives, this method, when combined with advanced bioinformatics, improves the sensitivity of sequence-based viral detection.

In this paper, we present a novel high-throughput application for a target-sequence enrichment approach using previously custom-designed baits. Our study demonstrates the ability to detect emerging variants, of low or high frequency (according to percentage of mapped reads), demonstrating the practical utility of this approach through the simultaneous analysis of up to ten pooled samples. This innovative pooling application was used for patient samples, which were detected positive for COVID-19, from various geographical locations, in order to characterize intra- and inter-patient viral variation. Our findings suggest that this NGS approach stands as a straightforward and valuable tool for routine analysis. Its efficiency allows for the early detection of SARS-CoV-2 variants and potential VOCs, contributing to a more effective response to the virus’s evolution. This approach also has notable advantages in terms of cost-effectiveness, making it a great choice for widespread implementation. Additionally, its minimal hands-on time further enhances its practicality, presenting a user-friendly solution for timely and resource-efficient analysis. Thus, this multifaceted approach is a comprehensive and accessible tool for monitoring SARS-CoV-2 evolution in routine analyses.

## 2. Materials and Methods

### 2.1. Patient Sample Collection and Preparation

For this study, nasal swabs were collected from 116 different patients. Additionally, we included 20 gargling samples with saline solution. Sampling was performed between May 2020 and March 2021 and in accordance with clinical practice guidelines at collection centers. Samples were collected at three different clinical sites as follows: Austria (AT; *n* = 36), Bosnia and Herzegovina (BIH; *n* = 70), and Portugal (PT; *n* = 30). All samples tested positive in routine SARS-CoV-2 qPCR, and after routine testing in the country of origin, the samples were shipped to the Medical University of Vienna where the subsequent sample preparation was performed. For sample preparation, 16 samples were processed individually (6 samples from Austria and 10 from Bosnia and Herzegovina), while the remaining samples were pooled in 12 pools of 10 samples each. Samples were pooled by country of origin (three pools from AT, six from BIH, and three from PT), time of collection, and collection method (ten pools of nasal swabs and two pools of saline gargling; see Table 1 and Table S1). For the single samples, 250 µL of each sample was pre-diluted 1:5 in phosphate-buffered saline (PBS) (pH 7; Dulbecco’s PBS, no calcium, no magnesium; Thermo Fisher Scientific, Waltham, Massachusetts, USA). On the other hand, pools were generated using a volume of 100 μL per sample (10 samples per pool) and the subsequent dilution of the pooled samples 1:5 in PBS. PBS, a water-based physiologic salt solution, facilitates the stabilization of intact virions for downstream viral particle purification steps. As gargling samples were collected directly in a physiologic saline solution (NaCl 0.9%), the samples were not pre-diluted.

### 2.2. Viral Particle Purification, RNA Extraction, and Virus Characterization

Purification of intact virus particles was performed by applying the Virus Purification and Enrichment Procedure (VIPEP), as described previously, with minor adaptions [7]. Briefly, virus particles were initially resuspended in PBS to increase virion stability (as mentioned above). Cellular remnants and cell debris were eliminated by two subsequent centrifugation steps and filtration through 0.45 μm pore size syringe filter units. Virus particles were purified by ultrafiltration using 50 kDa cut-off ultrafiltration devices (Amicon Ultra-15, Merck, Darmstadt, Germany). Free DNA was eliminated by DNase I treatment. Viral RNA was then extracted using the QIAamp Viral RNA Mini kit (Qiagen, Hilden, Germany). For rRNA depletion, we used a set of five specific rRNA-blocking oligonucleotides containing a 39-dideoxy C6 amino modification, as previously described [8]. cDNA synthesis was performed using SuperScript III (Thermofisher, Waltham, MA, USA) with a set of non rRNA binding hexamers to eliminate rRNA sequences further. The final amplification of total nucleic acids was performed by multiple displacement amplification (MDA) using a Repli-g kit (Qiagen, Hilden, Germany). Second-strand synthesis was performed using the NEBNext second-strand synthesis module (New England Biolab, Ipswich, MA, USA). Before commencing the target enrichment protocol, isolated viral nucleic acids were cleaned in a final purification step using Ampure Beads XP (Beckman Coulter, Brea, CA, USA).

### 2.3. Hybrid Capture-Based Target Enrichment, Library Preparation, and Next-Generation Sequencing

We used a commercially available bead-based target enrichment platform (Sure Select XT HS, Agilent, Santa Clara, CA, USA). The bait panel was designed by J. Breuer and colleagues and was made available upon request from the manufacturing company (Agilent, Santa Clara, CA, USA). The panel consists of approximately 50,000 bait sequences covering 813 full-length coronavirus genomes including 180 different SARS-CoV-2 sequences. Quality control of MDA-amplified cDNA was performed on a BioAnalyzer Platform (2100 Bioanalyzer Platform, Agilent, Santa Clara, CA, USA) in several steps throughout the enrichment protocol as follows: before and after fragmentation, after target enrichment, and after library preparation. Ultrasonic fragmentation of cDNA was performed using a Covaris S220 focused ultrasonicator (Covaris, Woburn, MA, USA) with a 5% duty factor, 200 cycles per burst, and a 120 s treatment time per sample. Target enrichment was performed as described in the application manual from the manufacturer (version 2019) with minor adaptions as follows: the input amount was between 50 and 100 ng cDNA, no carrier RNA or DNA was added, pre-capture PCR was run with 10 cycles (using index primers), and post-capture PCR was run with 18 cycles. Before sequencing, final quality control on the BioAnalyzer Platform was performed. Libraries were sequenced on an Illumina MiSeq (V3 Kit) sequencing platform in the 2 × 300 bp configuration.

### 2.4. Variant Calling, Data Filtering, and Spike Protein Variant Detection

After streamlining the sample workup, we adopted sequencing conditions to the manufacturer’s requirements and established means for bioinformatic data processing. In short, metagenomic raw sequencing quality was evaluated by using FASTQC 0.11.4 (http://www.bioinformatics.babraham.ac.uk/projects/fastqc/; accessed on 1 October 2021). Afterward, Trimmomatic 0.35 was used for primer sequences trimming and filtering of low-quality base calls [9]. Subsequently, the reads were mapped to the SARS-CoV-2 Wuhan-Hu-1 complete reference genome sequence (NCBI accession number: NC_045512.2) by using the software Bowtie2 2.2.7 [10]. SAM-Tools 0.1.19 [11] was used for alignment, and VarScan v2.3.9 as a variant caller for the detection of nucleotide substitutions, insertions, and deletions (http://varscan.sourceforge.net; accessed on 10 October 2021). As a last step, SnpEff 4.270 [12] was applied to detect alterations causing amino acid substitutions or other variants. Finally, visual validation of the mutations in the assembly files was performed to exclude bias variants with UGENE v. 42.0 software [13]. To rule out that occasionally occurring sequencing artefacts might be misinterpreted as nucleotide variants, we set a relative (10% of all reads at a single position) and absolute (10 reads per position) threshold level. Nucleotide variants supported by less than 10% of all reads at a single position or less than 10 reads in total were not used in this analysis. After recovering the SARS-CoV-2 mutations, we filtered the results specifically for the S-protein. These results were plotted in the form of a heat map with R v. 4.1.2 (R core team) using the package ComplexHeatmap V.2.8.0 [14]. Finally, to test for positive selection per gene, we used the software BUSTED v4.5 [15] using sequence alignments.

### 2.5. Primer Development and Optimization for Variant Confirmation

A sample pool (27B) from Austria was selected in order to single-sequence and confirm spike protein mutations detected within this pool with the target enrichment approach and thus validate our pooling method. Samples were freshly extracted with a Viral RNA Mini kit (Qiagen, Hilden, Germany) following the manufacturer’s protocol, and RNA degradation was assessed via RT-qPCR by using a LightMix^®^ SarbecoV for the detection of the E gene (Roche, Berlin, Germany). A new primer pair for PCR amplification was designed with Primer3Plus [16] to assess the sensitivity and assure the amplification of the detected variants, percentages of mapped reads that were not 100%, in this case, with 97.4% and lower, in one amplicon. This amplicon comprised four variants, including both indels and SNPs, with the percentage of reads of 63.6% (SNP at position 21,766—I68I), 70.6% (insertion at position 21,770—V70delinsAl), 94.3% (deletion at position 21,990—Y145del), and 97.4% (deletion at position 21,764—H69_V70del). To design these primers, the publicly available reference sequence (GenBank accession number: NC_045512.2) was used to locate the mutations and to search for conserved regions. A fragment of around 600 base pairs (bp) and primers with 18–22 bp, melting temperature of 52–58 °C, and a GC constitution of 40–60% were searched for. NetPrimer (Premier Biosoft International, Palo Alto, CA, USA) was used to verify if there were events of primer secondary structures. Detailed primer information and PCR conditions are shown in Table S2. Purified PCR products were sequenced using the standard Sanger sequencing protocol at Microsynth AG (Netherlands), using both forward and reverse primers. The software FinchTV 1.4.0 (Geospiza, Inc., Seattle, WA, USA; http://www.geospiza.com, accessed on 9 March 2022) was used to view DNA sequence chromatogram data, and the sequences were then imported and aligned against the ref-seq mentioned above using BioEdit version 7.2.5 [17].

## 3. Results

### 3.1. Sequencing Efficiency and Sequence Diversity

In this study, we looked at SARS-CoV-2 genomic sequence diversity in nasal swab and mouthwash samples from patients suffering from COVID-19. Sample specimens were collected from different geographic regions in Austria, Bosnia and Herzegovina, and Portugal during sequential waves of SARS-CoV-2 infections, between May 2020 and March 2021. We used a previously published protocol to enrich viral RNA genome sequences while removing bacterial and human-derived nucleic acid sequences to improve detection sensitivity. Following that, we used a custom-designed hybrid capture-based target enrichment assay prior to Illumina-based NGS to generate coronavirus-specific metagenomes. For that, we analyzed 16 individual patient samples separately (01A–16A) and pooled 120 samples into pools of 10 samples each, generating a total of 12 pools (25B–38B). NGS was highly efficient, generating a mean number of 1.0 × 10^6^ reads per sample that aligned to the SARS-CoV-2 reference genome NC_045512.2 (average of 3.1 × 10^5^ reads for single samples and 2.0 × 10^6^ reads for pools). Accordingly, the reads were mapped to the reference genome with an average sequence coverage of 96% of the total virus genome per sample/pool (96% for single samples and 95% for pools; full results are shown in Table 2 and Table S3), with very little human and bacterial DNA detected, confirming the target enrichment efficiency.

Overall, we detected a high level of metagenomic SARS-CoV-2 sequence diversity with a total of 624 mutations in 10 distinct SARS-CoV-2 gene regions, for all samples and pools. When analyzing all samples, the highest number of mutations was detected in ORF1ab (*n* = 323) followed by the spike (S) gene (*n* = 122) (Table 3). However, taking into account the number of unique mutations and gene size, we detected an average number of 8.2 mutations per 1 kb. The mutation rate was highest in the Envelope (E) gene region (52.6 mutations per 1 kb) followed by the Nucleocapsid (N) gene region (23.8 per 1 kb) (Table 3). On the other hand, ORF 7b and ORF 10 only presented one unique mutation per gene, and no mutation was present for gene ORF6.

**Figure 1:**
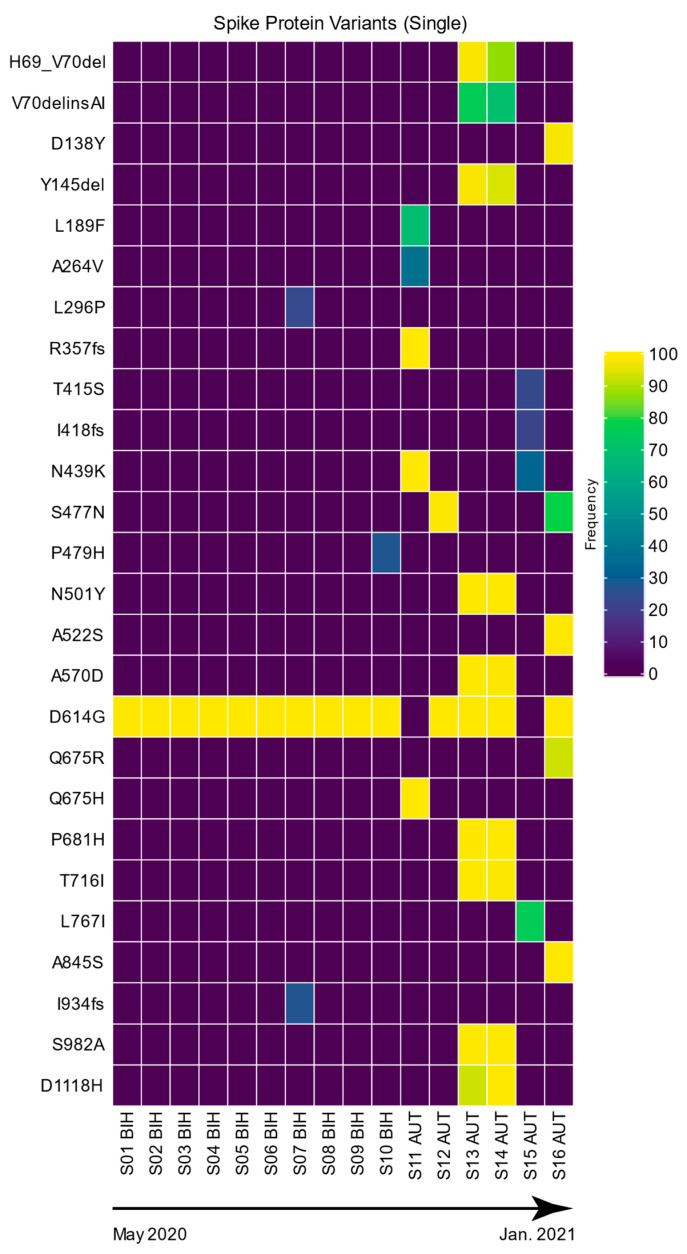
Spike protein mutation and corresponding frequencies (percentage of mapped reads) detected in the single samples. The frequencies of the variants are represented with a color gradient. The 16 single-sequenced samples are represented with the country codes. Dates represent the sampling collection period.

**Figure 2:**
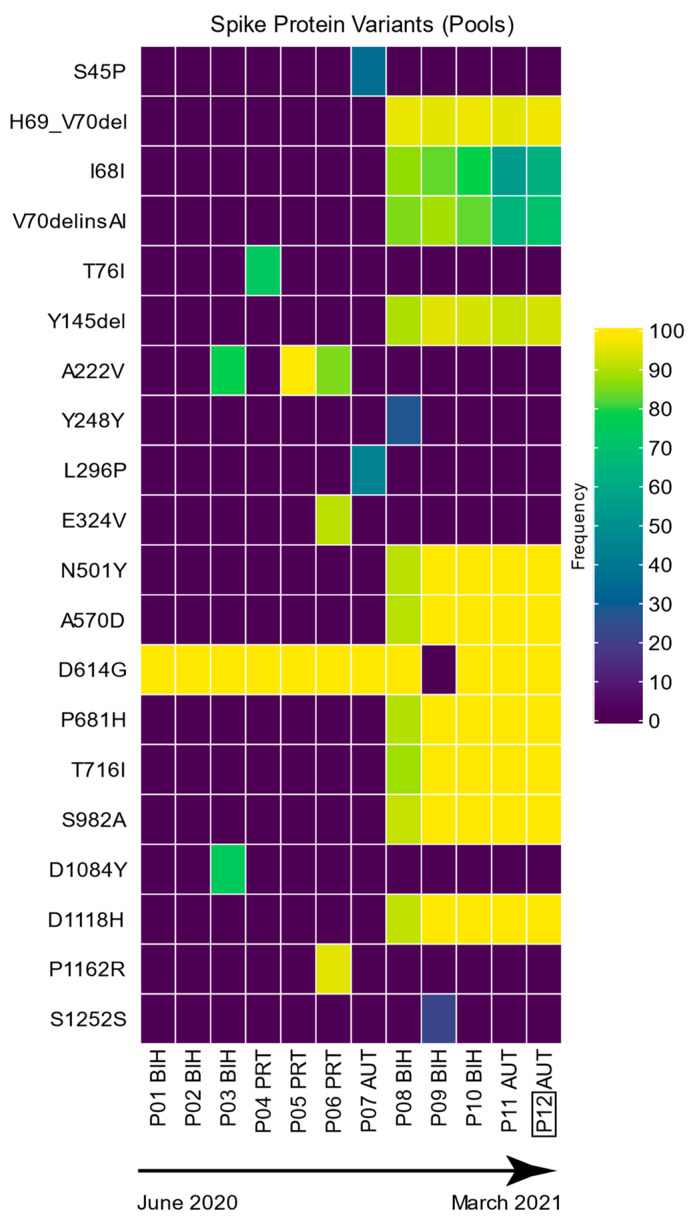
Spike protein mutation and corresponding frequencies (percentage of mapped reads) detected in the pooled samples. The frequencies of the variants are represented with a color gradient. The 12 pools of 10 samples each are represented with the country codes. P12 was the selected pool for assessing the sensibility of the mutations detected with NGS using PCR amplification. Dates represent the sampling collection period.

Specifically for the spike protein, from the overall 122 mutations that were identified for the S-gene, we found 35 different (unique) mutations, each causing a change in the spike protein amino acid sequence, with a detected mutation rate of 9.2 mutations per 1000 nucleotides (Table 3, Figure 1 and Figure 2). Because of its importance in virus replication and immunogenicity, we focused our subsequent research on the spike protein gene. Additionally, in the context of viral evolution, we inferred whether we could detect any positive selection. For this, we statistically tested the presence of positive selection per gene using the three genes showing the highest mutation rate (Envelope, Nucleocapsid, ORF8) as well as the spike gene. We took advantage of the Branch-site Unrestricted Statistical Test for Episodic Diversification analysis, and based on the likelihood ratio test, there was evidence of episodic diversifying selection for the spike gene (*p* = 0.0016; with four sites with Evidence Ratios (ERs) ≥ 10 for positive selection) and for the Nucleocapsid gene (*p* = 0.022; with three sites with ER ≥ 10 for positive selection). Nevertheless, there was no evidence of episodic diversifying selection for the Envelope (*p* = 0.18), or ORF8 (*p* = 0.47) genes, showing no sites with ER ≥ 10 for positive selection.

### 3.2. Spike Protein Variants—Single Samples

From analyzing SARS-CoV-2 metagenomic sequences derived from single-patient samples, we observed a high intra-patient sequence variability (see Figure 1). While most variants were supported by nearly 100% of the mapped reads (frequency) at individual positions, some mutations were supported only at lower persistence rates. We detected six mutations that showed intermediate sequence variability with 40–90% frequency (mapped reads). Another seven positions showed high sequence variability with frequencies between 20 and 40% within individual patients. Besides detecting intra-patient sequence variability, we also detected sequence variability between individuals. While some mutations occurred only occasionally, others were found at much higher frequencies (Figure 1). Mutation D614G, for example, was present in 14 out of 16 patients at a persistence rate of more than 99.8% of the total reads covering this position. Interestingly, a distinct pattern is also visible in two single samples from Austria (S13 and S14; Figure 1), easily identifying the Alpha variant (known as 20B/501Y.V1, VOC 20201s2/01, or B.1.1.7 lineage) by the combination of mutations HV69-70del, Y144del, N501Y, A570D, D614G, P681H, T716I, S982A, and D1118H, with the addition of I68I and V70delinsAI in all of these single samples. The mutation N439K is common to both single samples from Austria S11 and S15, and sample 12A had two mutations, and these are in common with sample S16 (S477N and D614G; Figure 1). Moreover, the Austrian sample 16A had several mutations mirroring the lineage B.1.1.317 (PMVL-43, S: D138Y, S477N, A522S, D614G, Q675R, A845S; hCoV-19/Russia/MOW-PMVL-43/2021).

### 3.3. Spike Protein Variants—Pooled Samples

We also detected sequence variability between pools. The D614G mutation was detected in 11 out of 12 sample pools. For two pools (P01 and P02), the D614G mutation was the only variation detected in the S protein coding sequence compared with the reference genome. These two pools also include the earliest collected samples (June 2020). In all D614G-positive pools, the mutation was supported by more than 99% of reads, indicating a high degree of sequence homogeneity at this position among the pooled samples. Six mutations were supported by 40–90% of reads and three with frequencies between 20 and 40% of the mapped reads per pool. This indicates that the pooled and single samples showed sequence heterogeneity at these S-gene positions. Similar to the single samples, a clear pattern was also visible in five pools (two from AT—P11 and P12 three from BIH P8-10; Figure 2) easily identifying the Alpha variant (known as 20B/501Y.V1, VOC 20201s2/01, or B.1.1.7 lineage) by the combination of mutations HV69-70del, Y144del, N501Y, A570D, D614G, P681H, T716I, S982A, and D1118H, with the addition of I68I and V70delinsAI. On the sample pools P03 (35B), P05 (37B), and P06 (38B), within the several mutations that were detected in the samples, two stabilizing mutations (D614G and A222V) belonging to the variant B.1 were also present.

### 3.4. Variant Confirmation with PCR

**Figure 3:**
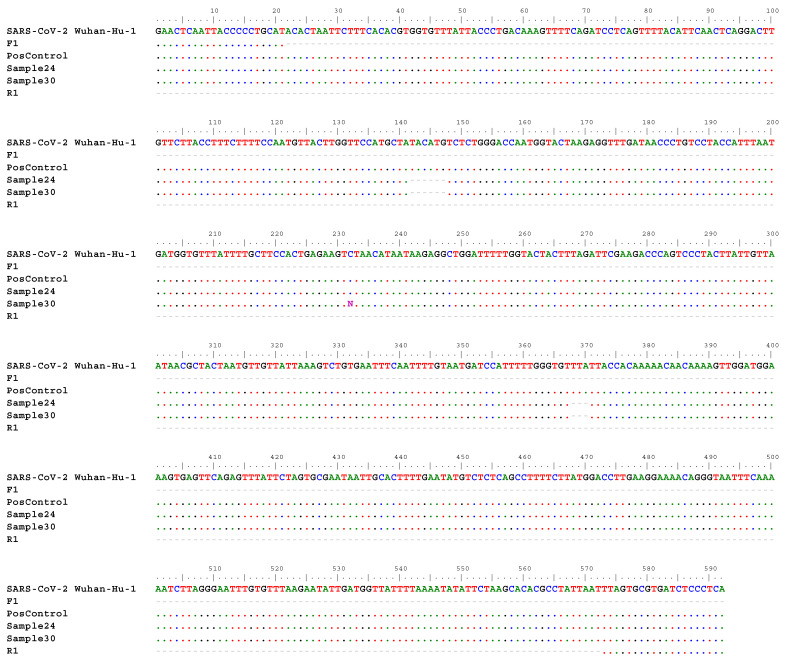
Nucleotide sequences where deletions were observed after PCR, confirming variants that were present in the pool and previously detected with NGS. In the alignment, the reference genome (SAR-CoV-2 Wuhan-Hu-1), forward primer (F1), positive control (PosControl), Sample 24, Sample 30, and reverse primer (R1) sequences are included.

Finally, to confirm the sensitivity of our method and ensure the amplification of the detected variants with NGS with frequencies that were not 100%, we exemplarily used pool P12 (27B) for variant amplification in the single samples that comprised that specific pool, using PCR and Sanger sequencing. For that, we designed a primer pair to amplify the mutations with frequencies less than 100%, in this case 97.4% and lower. This 592-base pair amplicon contains four distinct mutations, including three indels and one SNP. As for RNA degradation, we were only able to recover PCR signals for two of the ten samples from the pool (samples 24 and 30; Figure 3). Nevertheless, the Sanger sequencing results of PCR amplicons corroborated our NGS-derived sequence information from pooled samples. Nonetheless, for both samples, we detected two deletions—on position 21,764 on the reference genome (position 141 on the alignment; REF: ATACATG; ALT: A (frequency of 97.4%); Figure 3) and on position 21,990 on the reference genome (position 367 on the alignment; REF: TTTA; ALT: T (frequency of 94.3%; Figure 3).

## 4. Discussion

The “Red Queen hypothesis” [18] proposes that species must constantly adapt, evolve, and multiply in order to survive in the face of constantly evolving conflicting species. Moreover, the parasite (virus) and host (human) engage in an evolutionary arms race [1], which can result in positive selection of their traits related to fitness and survival via mutations. Whole genome sequencing, or at least complete or partial S-gene sequencing, is the best method for characterizing a specific SARS-CoV-2 variant, according to the European Centre for Disease Prevention and Control (www.ecdc.europa.eu/en, accessed on 3 December 2023). This means that adequate sample collection and diagnostic method selection are key factors in the successful implementation of the diagnostic testing strategy.

In this work, we present a novel high-throughput pooling approach for sequence-independent genome sequencing to detect viral variants effectively. We used a SARS-CoV-2 target enrichment approach to capture and enrich SARS-CoV-2 viral sequences effectively in different patient samples. This method is efficient even in samples with relatively low viral particle concentrations [19]. Nonetheless, by pooling samples and having access to mutations present in 10 times more genomes, we aimed at developing a more high-throughput, sensitive method. After recovering the SARS-CoV-2 mutations, we narrowed the results down to mutations specifically for the S-protein because of its known relevance.

### 4.1. Observed SARS-CoV-2 Mutation Rate

We target-sequenced SARS-CoV-2-positive samples from three distinct countries, in single samples or in pools of 10 samples each, and we were able to pool at least 10 samples to detect variants in different frequencies (percentage of mapped reads). For both approaches, our analysis suggests that our high-throughput sequencing-based methodology is highly sensitive for the detection of SARS-CoV-2, detecting from a low to high prevalence of mutations. Moreover, we were able to identify specific virus variants by detecting characteristic mutation patterns through the complete viral genome.

In this work, we compared our samples against the reference genome hCoV-19/Wuhan-Hu-1/2019. Our sample collection spans approximately one year, from May 2020 to March 2021. When comparing our samples’ collection time with the reference sequence Wuhan-Hu-1 (collected in December 2019), the total timespan is estimated to be less than 1.5 years. Within this period, we identified 624 mutations across the 11 viral genes in our 120 samples, with 245 being unique mutations.

A comparative analysis with the current literature reveals interesting insights. Previous studies report mutation rates ranging from around 23.6 mutations per year that are identified in SARS-CoV-2 sequences [20] to estimates of a mutation rate of 33 genomic mutations/year [21], both of which are lower than our observed number of unique mutations. On the other hand, Gálvez et al. [22] calculated a mutation profile for the SARS-CoV-2 genome from 386 samples in the first year of the pandemic in Colombia, detecting a total of 1662 mutations across the 11 viral genes. Considering the larger sample size of this study (approximately three times more samples), when adjusted (1662/3 = 554), the number of mutations becomes comparable to our observations of 624 total mutations in our study.

We observed an average mutation rate of 8.2 per 1 kb through the genome. However, when looking per gene, ORF 7b and ORF 10 only presented one unique mutation per gene, and ORF6 presented no mutation. Previously, Hassan et al. [23] found similar results when they discovered that the total number of mutations in three accessory proteins ORF6, ORF7b, and ORF10 was around 1.5%. Because these mutations are few, they hypothesized that this is because these mutated protein variants are beneficial to the virus.

Interestingly, Grigoriev [24] observed that mutational patterns in the SARS-CoV genome were much different from other coronaviruses in terms of mutation rates. The cause is probably not in the host as other human coronaviruses did not appear to be different from the other viruses examined and because the mutation rates were in general agreement with the model of the coronavirus lifecycle.

It is important to note that the calculated mutation rates in this work are specific to the samples used during this period and may not reflect mutation rates observed in subsequent periods, with more recent viral variants. Moreover, the identification and characterization of more complex variants, such as Omicron, may pose additional challenges in distinguishing among different viral strains. These considerations underscore the dynamic nature of viral evolution and the importance of ongoing research efforts in this field.

### 4.2. Intra- and Inter-Patient SARS-CoV-2 Sequence Variability

Interestingly, when analyzing the single sequenced samples, we discovered intra-patient variation, as for several patients the percentage of mapped reads for certain mutations was not close to 100%, reaching as low as 22%. In the literature, most studies focused on inter-patient diversity assuming that only one virus variant infects each patient. Despite this, because of the high mutation rates of RNA viruses, it can be assumed that patients carry a diversity of virus quasispecies [25]. Previously, minority viral populations (up to 1%) were observed during the course of SARS-CoV-2 infection, where quasispecies differed from one day to the next, or even among anatomical sites in the same patient [26]. This suggests that in vivo, this type of coronavirus appears to be complex and dynamic concerning variants. Thus, from whole-genome deep sequencing raw data, intra-host composition analysis of minor variants and the amount of intra-host genomic diversity in SARS-CoV-2 samples can be retrieved [27,28].

The sample pooling approach, on the other hand, appears to be a great strategy for the future rapid and cost-efficient clinical screening of newly emerging variants, with the precision and sensitivity of NGS. At the same time, our plotting in the form of a heatmap is very visual, not only easily showing the percentage of mapped reads (frequency) of the mutations but also easily detecting which mutations the different samples have (or not) in common, deciphering the SARS-CoV-2 variants that are present. Furthermore, by examining the mutation heatmaps (Figure 1 and Figure 2), a clear pattern was visible in two single samples and five pools, easily identifying the Alpha variant by the combination of mutations. This variant emerged in September 2020 in the U.K., and our samples were collected from Austria and Bosnia and Herzegovina, between January and March 2021. Even though our pooling method was applied with samples from the same country, when no patient tracking is required, such as in this case, other relevant sample combinations, such as pooling samples from patients with similar disease states or organizing groups from low to high severity, can be made and compared using our pooling method.

It is possible to observe single nucleotide variants (SNVs) at low percentages in individual samples (Figure 1). Upon closer examination, SNVs at low percentages are found to be unique to each singular sample, suggesting a minimal risk of contamination and rather co-infection of different variants. Similarly, by observing the presence of SNVs at low percentages in the pooled samples (Figure 2), it is reasonable to assume the possible presence of different variants within the different pooled samples or even co-infections because of the pooling nature of the samples. Moreover, given the separate processing and sequencing of single and pooled samples, which occurred in different months, we are confident that any observed variants likely arise from co-infections in single samples or mixtures of different variants or co-infections in pooled samples. Interestingly, Pipek and colleagues [29] investigated a database containing the raw sequencing data of more than 2 million SARS-CoV-2 samples and identified 0.35% of them as co-infection cases. They further set out to detect the presence of intra-host recombinants and showed that a threshold of 0.1 for the ratio of recombinant reads overlapping a given position might be reasonable to get rid of PCR-induced artifacts.

Still, one potential drawback of the pooling method could be that if a single low-frequency mutation is present in only one sample, the mutation may go undetected, which is not a limitation when single-sequenced. This low-frequency mutation might go unnoticed because the number of reads in a pool is approximately ten times greater than the number of reads in single sequencing. For example, if a mutation has 30% frequency (percentage of mapped reads) and is found in only one individual from a pool of ten samples, it would have a very low frequency in the pool and therefore would go undetected. However, even if this scenario occurs, it should not necessarily be a cause for concern. While it is true that some rare variants may be missed, including those that never become dominant, ongoing surveillance efforts can still detect emerging mutations as they begin to increase in frequency across a greater number of samples. Furthermore, nowadays, NGS has evolved into a routine application, being a considerably less resource-intensive method for both wet and dry labs, when compared with a few years ago. Genomics is the most effective technique for monitoring and discovering novel variations (which could become VOCs), and our method fits flawlessly. As a result, high throughput methods enable rapid public health responses (such as contact tracing) and real-time estimates of the prevalence of specific variants in the community. Additionally, reductions in sensitivity or failure to detect the circulating or emerging variants caused by mismatches in primer/probe sequences will not be detected, which is a significant advantage. Ultimately, our methodology is intricately cost-effective. With the potential for a cost reduction ratio of 1:10, exemplified by strategies such as employing 10 pools of 10 samples each, it becomes feasible to thoroughly assess the current epidemiological landscape in different countries concerning mutations.

### 4.3. Detection Analysis of SARS-CoV-2 Mutants—Spike Protein Example

The coronavirus spike protein mediates receptor binding and fusion of the viral and cellular membranes. Several fine-scale sequence variation analyses of SARS-CoV-2 isolates identified several genomic regions with increased genetic variation, including this region. As the number of mutations on the spike protein increases, new VOCs with varying viral characteristics such as severity, immunity, or infectivity emerge. Delta, for example, has been reported to slightly increase disease severity with higher hospital admissions when compared with the alpha VOC, while Omicron and Delta have higher viral load than other variants identified to date, which likely contributes to its inherently higher transmissibility (see [30,31]). Because of its importance and greater genetic variety, we used the spike protein region as an example, for simplicity.

With our novel method, we were able to detect several mutations, previously found within the GISAID (www.gisaid.org; accessed on 3 December 2023) database that were specific to single patient samples or pools, at times at very low frequencies, demonstrating that our method is effective in detecting low-frequency variants on the spike protein. With these results, we can demonstrate that we could equally detect new emerging VOCs very early in time when their frequency is still at low levels, and even at an intra-patient variation level.

In general, selection pressures may favor the emergence of variants that escape neutralizing antibodies in regions with high rates of transmission. Our findings support the significance of the D614G mutation, a prevalent alteration in the spike protein observed globally, which may provide advantages to the virus. This mutation, speculated to have originated in Europe in January 2020 (EPI_ISl_422424) from the lineage B.1, has become dominant worldwide [32]. Our data consistently detected the D614G mutation in most samples, emphasizing its prevalence, both in the pooled and single samples. Positioned in potential epitope regions (codon 469), D614G is implicated in enhancing infectivity by promoting more efficient S protein assembly into the virion [33]. Although it does not seem to exacerbate the disease or aid vaccine evasion, its accumulation in D614G-bearing lineages could potentially affect the stability of the spike and therefore may influence the binding affinity toward the ACE2 receptor [32]. Notably, the emergence of the Omicron (B.1.1.529) VOC, identified post-our sampling period, rapidly spread, surpassing the previously dominant Delta VOC (B.1.617.2), further underscoring the role of mutations like D614G or N501Y in enhancing infectivity. During the first waves of the pandemic, with the rise in lineages B.1.351 and P.1, the mutation N501Y together with at least two other non-synonymous substitutions, K417N/T and E484K, have been found to confer escape from neutralizing antibodies (see [34]).

As the pandemic progressed, critical mutations affecting the protein structure of SARS-CoV-2 emerged, potentially influencing disease treatment and prevention approaches. Notably, the mutation N439K, identified in two samples from Austria, has demonstrated the capacity to modify infection efficiency and antigenicity based on molecular dynamics simulations [35]. First discovered in Scotland from lineage B.1 in March 2020, N439K has independently reoccurred multiple times [35]. Sample S11, resembling the B.1.258.17 sublineage of B.1.258∆ and anticipated to circulate in Central Europe during Fall 2020, shares this mutation and has accumulated additional substitutions in the S protein, including L189F [36]. Samples S11 and S15 share the mutation N439K. Conversely, sample S16 mirrors lineage B.1.1.317 (PMVL-43), exhibiting specific mutations (S: D138Y, S477N, A522S, D614G, Q675R, A845S; hCoV-19/Russia/MOW-PMVL-43/2021; https://virological.org/t/spread-of-endemic-sars-cov-2-lineages-in-russia/689 accessed on 30 January 2024; [37]), while sample pools P03, 05, and 06 harbor stabilizing B1.1 mutations D614G and A222V alongside other detected mutations [32]. This diversity in mutation profiles underscores the dynamic nature of SARS-CoV-2 evolution.

On the other hand, not only SNPs but also insertions/deletions (indels) are very common modifications in the evolution of viral genomes. Aside from the previously mentioned indels, which were found in multiple samples, we also detected three indels, which were present only one time in three different single-patient samples. The duplication I934fs and deletion I418fs, where both are present with a very low percentage of mapped reads in samples S07 (26.97%; BIH) and S15 (21.63%; Austria), respectively, showed intra-patient variation. On the other hand, R357fs is a deletion with a very high percentage of mapped reads (99.95%), which was present only in sample S11 from Austria. In addition, other recognized VOCs within the sampling period (until March 2021), such as the Beta (B.1.351—South African, December 2020) variant, were not detected—no K417N spike mutation present nor Gamma (P.1)—as no samples had either K417N or H655Y spike mutations present. Moreover, the Delta variant most likely emerged in May 2021, as it was not detected in our samples (Figure 1 and Figure 2). Indels occur most frequently in the spike protein, but they can also be found in other proteins, particularly those involved in interactions with the host immune system [38]. Indels, although understudied, can have beneficial evolutionary effects, as recurrent deletions in SARS-CoV-2 spike glycoprotein were shown to confer resistance to neutralizing antibodies [39]. Of these, a few common deletions identified in certain SARS-CoV-2 variants including VOC such as ΔH69/ΔV70 or S Δ144 (primarily found in U.K. variants) are very well described and demonstrated to have recurrent emergence and transmission [5,40]. Interestingly, Rao and his colleagues [40] discovered that more than half of the 1.79 million SARS-CoV-2 spike protein sequences contained at least one or more indels.

Specifically for the detected spike protein V70delinsAI insertion, it was observed a total of seven times in our data (two times in single samples and five times in pools). This variant involves the deletion of valine (V) at position 70 and the insertion of alanine (A) and isoleucine (I) at the same position. Garushyants et al. [41] conducted a survey of publicly available SARS-CoV-2 genomes to characterize potential insertion variants that may affect the virus’s pathogenicity during the COVID-19 pandemic. With their study, they concluded that the emergence of identical or similar insertions within the Delta variant background, potentially through recombination, could have significant epidemiological implications. Therefore, monitoring insertion variants is crucial, particularly with vaccination efforts that may drive the selection of escape variants [41].

## 5. Conclusions

NGS is a cost-efficient, high-throughput technology that has aided in the discovery of novel SARS-CoV-2 variants, evolution, and distribution patterns, all of which are crucial in the development of effective disease control and prevention methods. Initially, our intention was to include the 16 samples subjected to NGS as individual samples within the 12 pools. However, when the same 16 samples were pooled, we were unable to analyze the data because of sequencing failure. Consequently, we had to adapt our approach, taking into account challenges during the beginning of the COVID-19 pandemic. While we acknowledge this deviation from our original plan and recognize its implications, we believe that our findings remain valid and contribute to the understanding of variant detection methods. While we recognize the importance of sample size in research, it is crucial to acknowledge the limitations imposed by the challenging circumstances during the pandemic at the time of this study. Despite our efforts, the sample size of 136 samples in this study reflects the technical and logistical challenges encountered in obtaining positive samples from countries other than Austria at the time of data collection. Moreover, although we recognize the potential value of investigating intra-patient sequence variation signatures and their relationship with disease severity, access to this specific clinical information was not available for our study. Additionally, we were unable to assess any potential effects of vaccination on sequence diversity because of the lack of vaccination information.

In this work, we developed an NGS target enrichment-based approach that, despite its potential flaws, proves to be a very valuable strategy for genetic characterization, mutation screening, and virus evolution monitoring without the need for prior knowledge of the presence of specific mutations in the viral genome. As a result, when no patient tracking is required, our new pooling method is an excellent technique for detecting new variants before they become widespread. Our approach proved to be an effective strategy for accurately detecting and identifying newly emerging variants at a very early stage, including the prevalence calculation of VOCs. As a result, this method is versatile and applicable in other scenarios—it is highly adaptable to changes in the (local) epidemiological situation and available resources. In conclusion, our NGS method is of high importance, contributing to a future better understanding of emerging variants and for genetic characterization of the virus from both intra- and inter-patient variation, and it can serve as a model for other viral studies.

## Figures and Tables

**Table 1:** Sample information for the single and pooled samples.

Single Samples				Pooled Samples			
Name	Code	Country	Sampling Date	Name	Code	Country	Sampling Date
S01	01A	BIH	May 2020	P01	33B	BIH	June 2020
S02	02A	BIH	May 2020	P02	34B	BIH	June 2020
S03	03A	BIH	May 2020	P03	35B	BIH	June 2020
S04	04A	BIH	May 2020	P04	36B	PT	November/December 2020
S05	05A	BIH	May 2020	P05	37B	PT	November/December2020
S06	06A	BIH	May 2020	P06	38B	PT	November/December2020
S07	07A	BIH	May 2020	P07	28B	AUT	November/December2020
S08	08A	BIH	May 2020	P08	30B	BIH	January/February 2021
S09	09A	BIH	May 2020	P09	31B	BIH	February/March 2021
S10	10A	BIH	May 2020	P10	32B	BIH	February/March 2021
S11	11A	AUT	November 2020	P11	25B	AUT	February/March 2021
S12	12A	AUT	January 2021	P12	27B	AUT	February/March 2021
S13	13A	AUT	January 2021				
S14	14A	AUT	January 2021				
S15	15A	AUT	January 2021				
S16	16A	AUT	January 2021				

**Table 2:** Overall alignment rate, including percentage and number of sequences that aligned to the viral reference genome.

Overall Alignment Rate	Percentage	Number of Read Pairs
Average	96%	1,021,603
Single samples	96%	308,883
Pooled samples	95%	1,971,898
Average per sample in pool (/10)	-	197,190

**Table 3:** Overall number and distribution of the detected mutations per gene region, gene size, and mutation rate information for all samples. Genes are in the order as they appear on the genome.

Gene	Genome Position	Gene Size (nt)	Total N° Mutations Detected (N° mut)	Total N° Unique Mutations	Mutation Rate = (N° mut/nt). 1000
ORF1ab	266–21,555	21,290	323	139	6.5
Spike (S)	21,563–25,384	3822	122	35	9.2
ORF3a	25,393–26,220	828	21	8	9.7
Envelope (E)	26,245–26,472	228	23	12	52.6
Matrix (M)	26,523–27,191	669	10	8	12.0
ORF 6	27,202–27,387	186	0	0	0.0
ORF7a	27,394–27,759	366	5	4	10.9
ORF7b	27,756–27,887	132	1	1	7.6
ORF8	27,894–28,259	366	32	7	19.1
Nucleocapsid (N)	28,274–29,533	1260	84	30	23.8
ORF10	29,558–29,674	117	3	1	8.5
Total	0–29,903	29,903	624	245	8.2

## Data Availability

Raw metagenomics data were uploaded to NCBI (submit.ncbi.nlm.nih.gov; NCBI BioProject ID: PRJNA1071862) and will be publicly available upon manuscript publication.

## Supplementary Materials

The following supporting information can be downloaded at: https://www.mdpi.com/article/10.3390/v16050786/s1, Table S1: Sample description of the single (16) and pooled (12) samples; Table S2: PCR reaction mix and cycle information; Table S3: Raw data read filtering; Table S4: Mutations detected after data filtering, for the different viral genes.
